# Editorial: Ribosome survey and summary collection 2020

**DOI:** 10.1093/nar/gkz1217

**Published:** 2020-02-09

**Authors:** Christine M Dunham, William S Dynan, Alexander Mankin

**Affiliations:** 1 Department of Biochemistry, Emory University School of Medicine, Atlanta, GA 30322, USA; 2 Department of Radiation Oncology, Emory University School of Medicine, Atlanta, GA 30322, USA; 3 Center for Biomolecular Sciences, University of Illinois at Chicago, Chicago, IL 60607, USA

NAR is pleased to present a collection of six Survey and Summary articles highlighting important recent developments in the ribosome and translation field. The inspiration for the collection came from the most recent Ribosome meeting, held in January 2019 in Mérida, Mexico, where >250 researchers from 20 countries attended. These reviews highlight recent developments and techniques that move studies of the protein synthesis apparatus forward. The current collection reflects some, but by no means all, of the key topics that were discussed at the meeting.

One of the hot topics in the ribosome field that has emerged over the past few years is translation quality control. Cells invest large resources in rescuing ribosomes that get stuck while synthesizing proteins, helping ribosomes to bypass obstacles, and degrading damaged ribosomes and mRNAs. The mechanics of this quality control machinery in bacteria and eukaryotes have attracted considerable interest and experimental effort. One of the intriguing ideas is that the cell senses the ribosome traffic jam that arises when a trailing ribosome bumps into a stalled ribosome. Characterization of how colliding ribosomes are recognized and unraveling the subsequent events brings important insights into how ribosomes operate in the cell, as discussed in articles by Inada (1) and Collart and Weiss (2).

Another important area of contemporary research is ribosomopathies—alterations in the ribosome structure or biogenesis that underlie human diseases. Two related, but independent, subjects border this topic. One is the role of posttranscriptional rRNA modifications and post-translation modifications of ribosomal proteins. The functional importance of many of these modifications has intrigued researchers for decades, yet, for many of them, we still lack a fully satisfying explanation of their purpose. A second area of intense research and debate is whether there exist specialized populations of ribosomes involved in translation of specific types of mRNA. It has been known for some time that the bacterial and eukaryotic cellular ribosomal pools are not uniform: ribosomes may differ in their composition and chemical makeup. This, in turn, affects their structure and functionality. How and whether cells exploit this heterogeneity is discussed by De Keersmaecker *et al.* (3).

While structural methods like X-ray crystallography and cryo-EM have advanced the understanding of the ribosome structure, genome-wide approaches illuminate the ribosome in action in the living cell. Like the microscope of van Leeuwenhoek, which revealed the existence of the previously unknown world of microbes, ribosome profiling (Ribo-seq), which maps the position of translating ribosomes on mRNA, has illuminated many unknown aspects of translation. Ribo-seq provides an extraordinarily crisp picture of the gene expression landscape in the cell and has highlighted the existence of many previously unknown short and unrecognized protein-coding sequences translated by the ribosome. These ‘alternative’ genes have escaped computational predictions, but they encode an important, previously ‘dark’ side of the cellular proteome, as discussed in the article by Storz *et al.* (4).

Yet another way the ribosome can generate alternative gene products is by deviating from the conventional rules of translation that require the ribosome to read the genetic message in a strictly defined, codon-by-codon mode. Various manifestations of translational recoding, including stop codon bypass, programmed frameshifting, or even skipping of several codons, allow for synthesis of polypeptides whose sequences deviate from those that would be produced by conventional translation of the genetic message. New insights into the mechanics of recoding are discussed by Rodnina *et al.* (5).

While progress in our understanding of the general molecular mechanisms of translation has been amazing, there is one vexing problem, which so far has received only limited attention. This problem is the origin and evolution of protein synthesis, which is inseparable from the key question of biology: what is the origin of life? Although the primordial ribosome may well have been an RNA-only machine, this appealing idea still lacks convincing experimental support. The influence of many elegant hypotheses regarding the rise of genetically-coded protein synthesis will remain limited until their legitimacy is experimentally validated. Retro-evolution of the ribosome in an attempt to restore or reveal some of its primordial functions could be one approach for addressing the origin of the protein synthesis apparatus. New ribosome engineering attempts, including those discussed by Hammerling *et al.* (6) could provide some much-needed tools, but other approaches and fresh ideas are in high demand.
